# Plasmid DNA Production in Proteome-Reduced *Escherichia coli*

**DOI:** 10.3390/microorganisms8091444

**Published:** 2020-09-21

**Authors:** Mitzi de la Cruz, Elisa A. Ramírez, Juan-Carlos Sigala, José Utrilla, Alvaro R. Lara

**Affiliations:** 1 Departamento de Procesos y Tecnología, Universidad Autónoma Metropolitana-Cuajimalpa, Mexico City 05348, Mexico; witzi97@gmail.com (M.d.l.C.); ole.eli.ale@gmail.com (E.A.R.); jsigala@cua.uam.mx (J.-C.S.); 2 Systems and Synthetic Biology Program, Centro de Ciencias Genómicas, Universidad Nacional Autónoma de México, Cuernavaca 62210, Mexico; utrilla@ccg.unam.mx

**Keywords:** minimal cells, proteome reduction, lean proteome, plasmid DNA

## Abstract

The design of optimal cell factories requires engineering resource allocation for maximizing product synthesis. A recently developed method to maximize the saving in cell resources released 0.5% of the proteome of *Escherichia coli* by deleting only three transcription factors. We assessed the capacity for plasmid DNA (pDNA) production in the proteome-reduced strain in a mineral medium, lysogeny, and terrific broths. In all three cases, the pDNA yield from biomass was between 33 and 53% higher in the proteome-reduced than in its wild type strain. When cultured in fed-batch mode in shake-flask, the proteome-reduced strain produced 74.8 mg L^−1^ pDNA, which was four times greater than its wild-type strain. Nevertheless, the pDNA supercoiled fraction was less than 60% in all cases. Deletion of *recA* increased the pDNA yields in the wild type, but not in the proteome-reduced strain. Furthermore, *recA* mutants produced a higher fraction of supercoiled pDNA, compared to their parents. These results show that the novel proteome reduction approach is a promising starting point for the design of improved pDNA production hosts.

## 1. Introduction

The availability of production strains optimized for industrial bioprocesses is critical for successful commercialization of biotechnologies. Optimized strains ideally display decreased maintenance demand and metabolic burden, which should result in increased productivity without alteration of relevant physiological parameters [1,2]. Therefore, the development of minimal cells for bioprocessing is a very promising field. An interesting approach to develop minimal cells is the elimination of genome sections considered dispensable under bioprocess conditions [2]. Such so-called genome-reduction approach has been employed in *Escherichia coli* by several groups [3]. A different approach consists in the identification of proteins not essential under process conditions and removing the burden of their expression [4]. This would generate an optimal resource allocation that maximizes the designed cell function [5]. Lastiri and co-workers [6] developed a method to engineer the resource allocation of *Escherichia coli*. This method identifies the minimum combinatorial set of genetic interventions that maximizes resource savings, which they applied by removing transcription factors that activate the expression of unused functions with the greater proteomic load. Namely, the deletion of *phoB*, phosphate scavenging system; *flhC*, flagella master regulator, and *cueR*, copper efflux system, resulted in a 0.5% reduction of the proteome compared to the wild type strain [6]. The mutant strain, named PFC, displayed an increased proteomic budget and improved performance when expressing a synthetic pathway for violacein production. In the present contribution, the performance of the proteome-reduced PFC strain for plasmid DNA (pDNA) production was evaluated. pDNA is becoming attractive as a therapeutic agent [7]. For instance, there are currently four pDNA-based candidate vaccines against the coronavirus disease 2019 (COVID19) under clinical trials, whereas 12 more are under preclinical evaluation [8]. Therefore, it is highly desirable to develop robust hosts for industrial scale pDNA production are highly desirable. pDNA production in genome-reduced strains has been described [9]. However, there are, to the best of our knowledge, no reports on the use of proteome-reduced strains for this purpose. pUC57kan production in the proteome-reduced strain was studied in a mineral medium and two complex media widely used in small-scale cultures: lysogeny broth (LB) and terrific broth (TB). Fed-batch cultures in shake flasks were also carried out in order to test the strain under conditions closer to industrial scales.

## 2. Materials and Methods

### 2.1. Bacterial Strains and Plasmid

*E. coli* K-12 derivatives BW25113 was used as wild type. The combinatorial mutations of the PFC strain (BW25113 Δ*phoB*, Δ*flhC*, Δ*cueR*) developed by Lastiri and coworkers [6] were generated by sequential P1 phage transduction from the individual knockout using the Keio collection strain as donors. The *recA* gene was inactivated in both strains using the methodology proposed by Datsenko and Wanner [10]. A PCR product obtained from previous *recA*::*Cm* strain [11] was used to increase the *recA* homology region, and the primers 5′-AGTGAAGAGAAGCCTGTCGG-3′ and 5′-ACGCGCTCGTAATCTTCTGC-3′. The strains were transformed with plasmid pUC57kan (GenScript, NJ, USA) and preserved at −70 °C.

### 2.2. Batch Cultures in Shake Flasks

The strains were precultured in lysogenic broth Lennox (Sigma, St. Louis, NA, USA) for at 37 °C for 10 h. Aliquots was taken to inoculate 250 mL baffled shake flasks containing 50 mL of the corresponding medium. The initial absorbance was 0.3 for cultures in LB and TB media. The composition of the mineral medium (in g L^−1^) was: K_2_HPO_4_, 17; KH_2_PO_4_, 5.3; (NH_4_)_2_SO_4_, 2.5; NH_4_Cl, 1.0; Citrate-Na_3_·2H_2_O, 2; MgSO_4_·7H_2_O, 1.0; and Thiamine-HCl, 0.01. The medium was supplemented with 2 mL L^−1^ trace element solution, and 2.5 g L^−1^ glucose. The trace element solution composition (in g L^−1^) was: ZnCl_2_, 10.5; ethylenediaminetetraacetic acid (EDTA), 5.5; CoSO_4_·7H_2_O, 1.5; MnSO_4_·H_2_O, 6.4; CuSO_4_·5H_2_O, 1.1; H_3_BO_3_, 1.5; Na_2_MoO_4_·2H_2_O, 1; FeCl_3_·6H_2_O, 51.4; and Cit-H·H_2_O, 39.9. LB medium contained 10 g L^−1^ Tryptone, 5 g L^−1^ yeast extract, and 5 g L^−1^ NaCl. TB medium composition is (in g L^−1^): tryptone, 12; yeast extract, 24; K_2_HPO_4_, 9.4; KH_2_PO_4_, 2.2, and supplemented with 8 mL L^−1^ glycerol. All the chemicals were purchased from Sigma (St. Louis, USA). All media were supplemented with kanamycin sulfate (50 µg mL^−1^). The flasks were incubated at 37 °C and 250 rpm with an orbital diameter of 50 mm. Samples were withdrawn every hour for analyses. All the cultures were carried out in triplicate.

### 2.3. Fed-Batch Cultures in Shake-Flasks

Precultures were harvested to inoculate 500 mL baffled shake flasks containing 50 mL of EnPresso B Plasmid medium (EnPresso GmbH, Berlin, Germany) prepared as indicated by the manufacturer and supplemented with kanamycin sulfate (50 µg/mL). The flasks were incubated at 37 °C and 250 rpm with an orbital diameter of 50 mm. Samples were withdrawn every hour from 18 to 24 h of culture. Cultures were carried out in triplicate.

### 2.4. Analyses

Cell growth was followed by optical density at 600 nm measured in a Biophotometer (Eppendorf, Hamburg, Germany). Cell dry-weight was calculated multiplying by a predetermined factor of 0.35 g L^−1^ cell dry-weight per absorbance unit at 600 nm. To obtain this factor, samples were taken from 500 mL shake flasks triplicate cultures and absorbance at 600 nm was measured. Cell pellets were collected by centrifugation of 6 mL of the broth in 1.5 mL centrifuge tubes and washed with 0.1% NaCl solution. The pellets were dried at 80 °C for 30 h and cell dry-weight determined. The correlation factor was obtained from the slope of the plots of absorbance versus cell dry weights. Glucose was quantified by an enzymatic–electrochemical method in a YSI-2900 Biochemistry Analyzer (Yellow Spring Instruments, Yellow Springs, OH, USA). Approximately 2 mg of wet biomass were used for pDNA purification. Cell lysis and pDNA purification and extraction were carried out using a QIAprep Miniprep Kit (Qiagen, Hilden, Germany) according to the manufacturer’s recommendations. pDNA was eluted using 80 µL of TE buffer at 70 °C to enhance its recovery [12]. pDNA was quantified by UV spectroscopy in a Nanodrop 2000 spectrophotometer (Thermo Scientific, Walthman, MA, USA). pDNA supercoiled fraction (SCF) was determined by electrophoresis of 100 ng of pDNA in 1% agarose gel in TAE buffer for 1 h at 80 V. A sample of linear plasmid, obtained by digesting a sample with the enzyme *Bam*HI (Invitrogen, Carlsbad, CA, USA), was also loaded. Supercoiled pDNA was identified by the bands that migrate faster than the linear plasmid and correspond to the covalently closed circular (ccc) monomer [13]. Image analyses were performed with the Image J software (NIH, Bethesda, MD, USA). Therefore, the SCF here is referred to the ccc monomer. Other isoforms were not quantified.

### 2.5. Analyses

The specific growth rate (*μ*) was calculated by fitting the cell density over time to an exponential model during the exponential growth phase. pDNA yield from biomass (Y_pDNA/X_) was calculated by evaluating the slope of the plot of pDNA concentration against biomass concentration over the time period involved. Specific pDNA production rate (*q_pDN_*_A_) was calculated multiplying Y_pDNA/X_ by *μ.* Yields and rates in the batch cultures were calculated with over the exponential growth phase. For fed-batch cultures, yields and rates were calculated over the 18–24 h of culture. Statistically significant differences were determined by means of two-tails heteroscedastic *t*-Student tests with a confidence level of 90%. Significant differences were considered only for values of *p* < 0.1.

## 3. Results and Discussion

### 3.1. Batch Cultures

**Figure 1:**
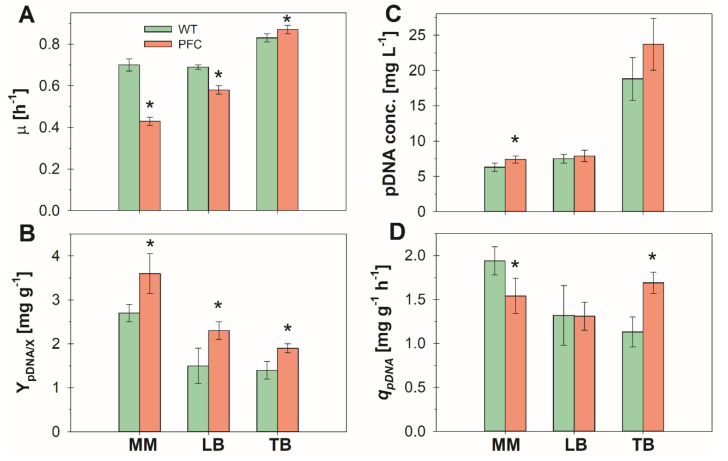
Main parameters of cultures of the wild type (green bars) and proteome reduced (PFC) (red bars) strains in mineral medium (MM), lysogeny (LB), and terrific (TB) broths. (**A**) Specific growth rate (*μ*) during the exponential growth phase. (**B**) Plasmid DNA (pDNA) yield on biomass (Y_pDNA/X_) during the exponential growth phase. (**C**) pDNA concentration at the end of the cultures. (**D**) Specific pDNA production rate (*q_pDNA_*) during the exponential growth phase. Error bars show the standard deviation between triplicate experiments. * indicates the groups where significant difference was found (*p* < 0.1).

Figure 1 shows the batch cultures performance in the different media. Time profiles of all cultures are shown in the Supplementary Material Figures S1–S4. The specific growth rate (*μ*) in exponential growth phase was lower for the PFC strain, in comparison with its wild type in mineral media and LB (Figure 1A). In the comparatively richer TB medium, *μ* was not significantly different for both strains. The pDNA yield from biomass (Y_pDNA/X_) was significantly higher for strain PFC than for its wild type in the different media (Figure 1B). The effect was more important in complex media. Namely, Y_pDNA/X_ for strain PFC was 33, 54, and 40% higher in mineral media, LB and TB, respectively, than for the wild type strain. This suggests that strain PFC can allocate more resources to pDNA synthesis in all the evaluated media, while the burden imposed by plasmid replication is efficiently compensated by the building blocks availability in TB, but not in the other media. The final pDNA concentration using the mineral medium was 18% higher in cultures of strain PFC, compared to its wild type (Figure 1C). Nevertheless, in the complex media, there were no significant differences in final pDNA concentration between strains (Figure 1C). Still, the fact that the same amount of pDNA can be produced with higher Y_pDNA/X_, and consequently less biomass, implies down-stream advantages. Despite the higher Y_pDNA/X_, the specific rate of pDNA production (*q_pDNA_*) in strain PFC was not significantly different than in its wild type in the mineral medium or LB (Figure 1D), which is a consequence of the decreased *μ* However, in the cultures using TB, *q_pDNA_* was 50% higher for the proteome-reduced strain in comparison with its wild type (Figure 1D).

The pDNA supercoiled fraction (SCF) is a relevant quality attribute for therapeutic applications. It has been recommended that for vaccination and gene therapy, pDNA should be supercoiled in at least 80% [14]. The achievable SCF depends, among other factors, on the host strain and vector size [13]. Agarose gel electrophoresis is widely used for topological analysis of pDNA. However, its resolution is very limited [12]. Figure S5 shows an example of the agarose gels obtained. The pDNA population identified as supercoiled correspond to those that migrated faster than the linearized plasmid and are most likely correspond to the ccc monomer [12].

**Figure 2:**
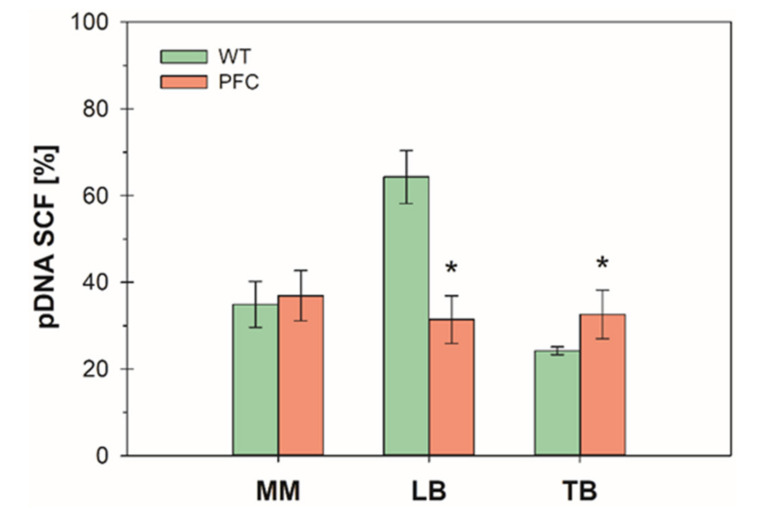
pDNA supercoiled fraction in cultures of the wild type (green bars) and proteome reduced (PFC) (red bars) strains in mineral medium (MM), lysogeny (LB), and terrific (TB) broths. Values correspond to sample taken at the end of the cultures. Error bars show the standard deviation between triplicate experiments. * indicates the groups where significant difference was found (*p* < 0.1).

Figure 2 shows the SCF of the pDNA produced by the wild type and PCF strains. The SCF was nearly the same for both strains when cultured in mineral media or TB. In contrast, when culture in LB, the SCF for the wild type strain was 2-fold higher than that of the proteome-reduced strain. Nevertheless, in all cases, the SCF was lower than 80%. Yau and coworkers [15] reported wide variation in the pDNA SCF produced in 17 *E. coli* strains. Nonetheless, the strain BW25113 was not included. Due its relevance, some strategies were explored with the aim of increasing SCF in the proteome-reduced strain, as described below.

### 3.2. Fed-Batch Cultures

There exist different strategies to increase pDNA supercoiling by engineering the vector, host, and culture. For instance, pUC57 was modified by inserting a strong gyrase binding site. This increased the supercoiling density and pDNA yield in strain DH5α [16]. The host can also be modified to increase the SCF. It has also been documented that deletion of the recombinase A (*recA*) gene substantially increased the pDNA SCF in *E. coli* W3110 and BL21 [11]. Fed-batch strategies at low dilution rates allows the manufacture of good quality pDNA at industrial scale [17,18]. Moreover, rationally designed amino acids supply can also improve pDNA quality [19]. Therefore, wild type and PCF Δ*recA* mutants were developed. All the four strains were cultured in a complex medium designed for attaining high cell-densities in shake flasks. That medium contains glucose as the main carbon source in the form of a polymer that is not degradable by *E. coli*. Glucose is released by the addition of a glucoamylase, thus controlling the growth rate and mimicking a fed-batch mode [20]. The version of such medium particularly designed for pDNA production greatly enhances Y_pDNA/X_ [21,22]. The use of purpose-designed medium and fed-batch mode better resemble the industrial conditions used for high-yield pDNA production [18].

**Figure 3:**
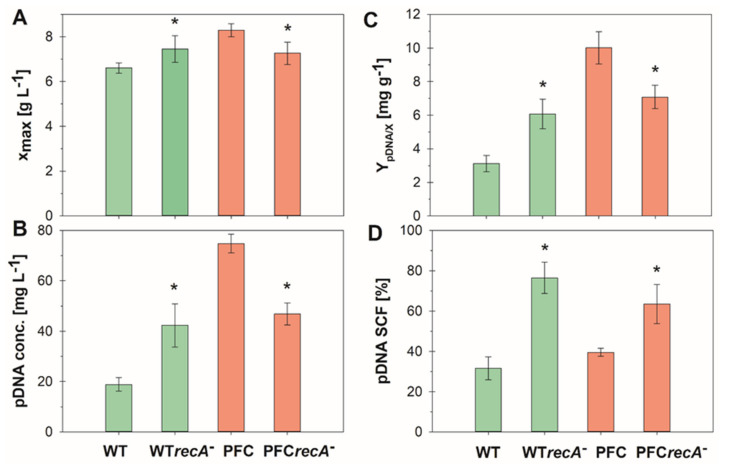
Main parameters of cultures of the wild type and proteome reduced (PFC) strains in fed-batch cultures in shake-flasks. Values represent the samples taken after 24 h of culture. (**A**) Maximum biomass concentration (x_max_). (**B**) pDNA concentration. (**C**) pDNA yield from biomass (Y_pDNA/X_). (**D**) pDNA supercoiled fraction (SFC). Error bars show the standard deviation between triplicate experiments. * indicates the groups where significant difference was found (*p* < 0.1).

The results of fed-batch cultures in shake flasks are summarized in Figure 3. All the strains reached more than 6 g L^−1^ biomass after 24 h of culture, and the highest cell-density (8.3 g L^−1^) was reached by strain PFC (Figure 3A). This is a good indicator of the increased resource allocation to pDNA production of proteome-reduced strain PFC under close to industrial conditions. The inactivation of *recA* caused only a slight decrease on the attained biomass for the wild type, while it decreased by 12% in the proteome reduced strain. The maximum pDNA concentration (75 mg L^−1^) was reached in strain PCF, which was four times greater than that of the wild type (Figure 3B). Interestingly, the inactivation of *recA* in the wild type resulted in a 2-fold pDNA production increase. In contrast, the same mutation reduced the amount of produced pDNA by 38% in the PFC strain (Figure 3B). Strain PFC reached a Y_pDNA/X_ of 10 ± 1 mg g^−1^, which was the highest of all the strains (Figure 3C), and 2.7-fold than the highest reached in batch cultures (Figure 1B). Similar to the effect on pDNA concentration, the inactivation of *recA* increased Y_pDNA/X_ almost 2-fold in the wild type strain, while decreased it by 27% in strain PFC (Figure 3C). The pDNA SCF was lower than 40% in the wild type and PFC strains (Figure 3D) but increased to 77 ± 8% and 64 ± 10% in the corresponding Δ*recA* mutants, respectively (Figure 3D). Jaén and coworkers [11] reported that *recA* inactivation in W3110 and BL21 increased the SCF but was accompanied by a reduction of Y_pDNA/X_ in both strains. This contrasts with previous results showing that the same mutation increased Y_pDNA/X_ in BL21(DE3) [23] and a W3110 derivative [24]. RecA protein stimulates the activity of topoisomerase A, a DNA-relaxing enzyme [25]. Consequently, *recA* mutants may display a lower topoisomerase I activity, which could contribute to the higher SCF observed. However, the impact of this mutation on Y_pDNA/X_ in dependence of the genetic background remains unclear.

Despite the attractive performance of the proteome-reduced strain, the Y_pDNA/X_ are still lower than those reported using thermal induction [26,27]. However, there is a number of additional genetic interventions that have been reported to enhance pDNA production [24,28,29].

## 4. Conclusions

Overall, the results described here show the potential of the proteome reduction approach to develop improved host production strains. Particularly for the case of pDNA production as an emerging biopharmaceutical, the proteome-reduced strain studied here, provided interesting results. The impact of proteome reduction on stress response to factors like bioreactor heterogeneities and heat induction are worth to be studied. Further cell engineering approaches addressed to increase pDNA production will be useful to improve the performance of the proteome-reduced strain.

## Supplementary Materials

The following are available online at https://www.mdpi.com/2076-2607/8/9/1444/s1, Figure S1. Time profiles of cultures of the wild type (left) and PFC (right) strains in mineral medium. Figure S2. Time profiles of cultures of the wild type (left) and PFC (right) strains in Lysogeny Broth. Figure S3. Time profiles of cultures of the wild type (left) and PFC (right) strains in Terrific Broth. Figure S4. Time profiles of cultures of the wild type (A), wild type Δ*recA* (B), PFC (C), and PFC Δ*recA* (D) strains in EnPresso B Plasmid medium. Figure S5. Example of agarose gel used for densitometric analysis of the pDNA produced by the proteome-reduced strain. L: pUC57Kan digested with BamHI; 1-3: pDNA samples from triplicate cultures in LB; 4-6: pDNA samples from triplicate cultures in mineral medium; and 7-9: pDNA samples from triplicate cultures in EnPresso B Plasmid medium.

## References

[1] Wu G., Yan Q., Jones J.A., Tang Y.J., Fong S.S., Koffas M.A.G. (2016). Metabolic Burden: Cornerstones in Synthetic Biology and Metabolic Engineering Applications. Trends Biotechnol..

[2] Ziegler M., Takors R., Lara A.R., Gosset G. (2020). Reduced and Minimal Cell Factories in Bioprocesses: Towards a Streamlined Chassis. Minimal Cells: Design, Construction, Biotechnological Applications.

[3] Kurokawa M., Ying B.W. (2020). Experimental challenges for reduced genomes: The cell model *Escherichia coli*. Microorganisms.

[4] Valgepea K., Peebo K., Adamberg K., Vilu R. (2015). Lean-proteome strains–next step in metabolic engineering. Front. Bioeng. Biotechnol..

[5] Hidalgo D., Utrilla J., Lara A.R., Gosset G. (2020). Resource Allocation Principles and Minimal Cell Design. Minimal Cells: Design, Construction, Biotechnological Applications.

[6] Lastiri-Pancardo G., Mercado-Hernández J.S., Kim J., Jiménez J.I., Utrilla J. (2020). A quantitative method for proteome reallocation using minimal regulatory interventions. Nature Chem. Biol..

[7] Ma C.C., Wang Z.L., Xu T., He Z.Y., Wei Y.Q. (2020). The approved gene therapy drugs worldwide: From 1998 to 2019. Biotechnol. Adv..

[8] World Health Organization (2020). DRAFT Landscape of COVID-19 Candidate Vaccines.

[9] Akeno Y., Ting B.W., Tsuru S., Yomo T. (2014). A reduced genome decreases the host carrying capacity for foreign DNA. Microb. Cell Fact..

[10] Datsenko K.A., Wanner B.L. (2000). One-step inactivation of chromosomal genes in *Escherichia coli* K-12 using PCR products. Proc. Natl. Acad. Sci. USA.

[11] Jaén K.E., Velazquez D., Delvigne F., Sigala J.C., Lara A.R. (2019). Engineering, *E. coli* for improved microaerobic pDNA production. Bioproc. Biosyst. Eng..

[12] Soto R., Caspeta L., Barrón B.L., Gosset G., Ramírez O.T., Lara A.R. (2011). High cell-density cultivation in batch mode for plasmid DNA vaccine production by a metabolically engineered *E. coli* strain with minimized overflow metabolism. Biochem. Eng. J..

[13] Schmidt T., Friehs K., Flaschel E., Schleef M. (2001). Structures of plasmid DNA. Plasmids for Therapy and Vaccination.

[14] Food and Drugs Administration of the United States of America (2007). Guidance for Industry: Considerations for Plasmid DNA Vaccines for Infectious Disease Indications.

[15] Yau S.Y., Keshavarz-Moore E., Ward J. (2008). Host strain influences on supercoiled plasmid DNA production in *Escherichia coli*: Implications for efficient design of large-scale processes. Biotechnol. Bioeng..

[16] Hassan S., Keshavarz-Moore E., Ward J. (2016). A cell engineering strategy to enhance supercoiled plasmid DNA production for gene therapy. Biotechnol. Bioeng..

[17] Rozkov A., Larsson B., Gillström S., Björnestedt R., Schmidt S.R. (2008). Large-scale production of endotoxin-free plasmids for transient expression in mammalian cell culture. Biotechnol. Bioeng..

[18] Williams J.A., Luke J., Langtry S., Anderson S., Hodgson C.P., Carnes A.E. (2009). Generic plasmid DNA production platform incorporating low metabolic burden seed-stock and fed-batch fermentation processes. Biotechnol. Bioeng..

[19] Dorward A., O’Kennedy R.O., Folarin O., Ward J.M., Keshavarz-Moore E. (2019). The role of amino acids in the amplification and quality of DNA vectors for industrial applications. Biotechnol. Prog..

[20] Panulla-Perällä S.J., Vasala A., Wilmanowski R., Casteleijn M.G., Neubauer P. (2009). Enzyme controlled glucose auto-delivery for high cell density cultivations in microplates and shake flasks. Microb. Cell Fact..

[21] Ramírez E.A., Velazquez D., Lara A.R. (2016). Enhancing plasmid DNA production in shake flask by enzyme-mediated glucose release and engineered *E. coli*. Biotechnol. Lett..

[22] Galindo J.E., Barrón B.L., Lara A.R. (2016). Plasmid DNA production in shake flasks is improved by enzyme-controlled glucose release. Ann. Microbiol..

[23] Phue J.N., Lee S.J., Trinh L., Shiloach J. (2008). Modified *Escherichia coli* B (BL21), a superior producer of plasmid DNA compared with *Escherichia coli* K (DH5α). Biotechnol. Bioeng..

[24] Borja M.G., Meza E., Gosset G., Ramírez O.T., Lara A.R. (2012). Engineering, *E. coli* to increase plasmid DNA production in high cell-density cultivations in batch mode. Microb. Cell Fact..

[25] Reckinger A.R., Jeong K.S., Khodursky A.B., Hiasa H. (2007). RecA can stimulate the relaxation activity of topoisomerase I: Molecular basis of topoisomerase-mediated genome-wide transcriptional responses in *Escherichia coli*. Nucleic. Acids Res..

[26] Carnes A.E., Luke J.M., Vincent J.M., Schukar A., Anderson S., Hodgson C.P., Williams J.A. (2011). Plasmid DNA fermentation strain and process-specific effects on vector yield, quality, and transgene expression. Biotechnol. Bioeng..

[27] Martins L.M., Oppolzer P.D., Sousa F., Queiroz J.A., Passarinha L.A. (2015). Enhanced biosynthesis of plasmid DNA from *Escherichia coli* VH33 using Box–Behnken design associated to aromatic amino acids pathway. Biochem. Eng. J..

[28] Gonçalves G.A.L., Bower D.M., Prazeres D.M.F., Monteiro G.A., Prather K.L.J. (2013). De novo creation of MG1655-derived *E. coli* strains specifically designed for plasmid DNA production. Appl. Microbiol. Biotechnol..

[29] Gonçalves G.A.L., Bower D.M., Prazeres D.M.F., Monteiro G.A., Prather K.L.J. (2012). Rational engineering of *Escherichia coli* strains for plasmid biopharmaceutical manufacturing. Biotechnol. J..

