# Supplementary material for: Plasmid DNA Production in Proteome-Reduced Escherichia coli
Source: Microorganisms. 2020 Sep 21;8(9):1444. doi: 10.3390/microorganisms8091444 (PMC7563601; doi:10.3390/microorganisms8091444)
Supplement: Supplementary file 1 [file microorganisms-08-01444-s001.pdf]

Supplementary Material to:

Plasmid DNA production in proteome-reduced *E. coli*

Mitzi de la Cruz, Elisa A. Ramírez, Juan-Carlos Sigala, José Utrilla, Alvaro R. Lara

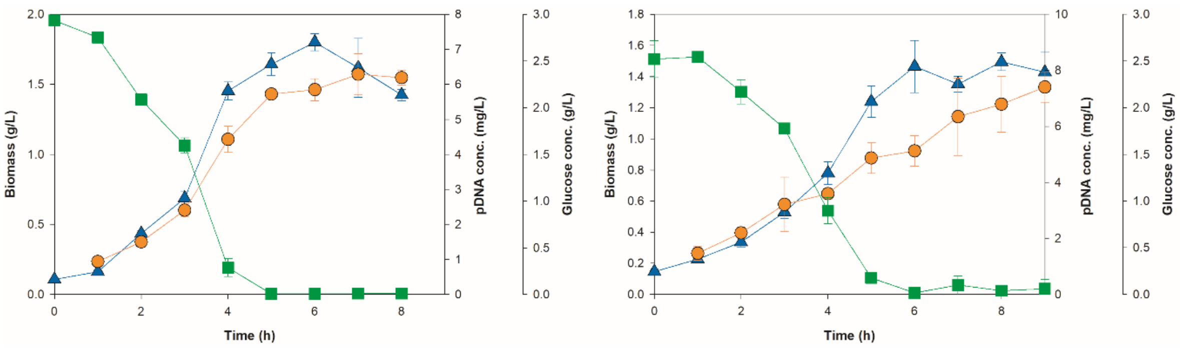

Figure S1. Time profiles of cultures of the wild type (left) and PFC (right) strains in mineral medium. Triangles: biomass; circles: plasmid DNA; and squares, glucose concentrations. Error bars show the standard deviation between triplicate experiments.

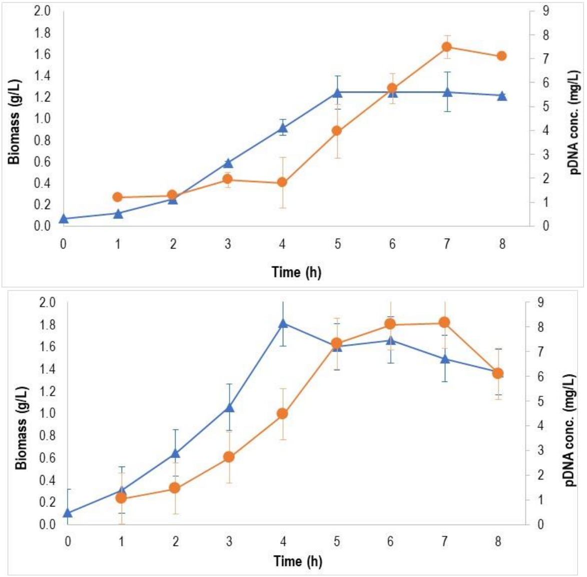

Figure S2. Time profiles of cultures of the wild type (left) and PFC (right) strains in Lysogeny Broth. Triangles: biomass; circles: plasmid DNA. Error bars show the standard deviation between triplicate experiments.

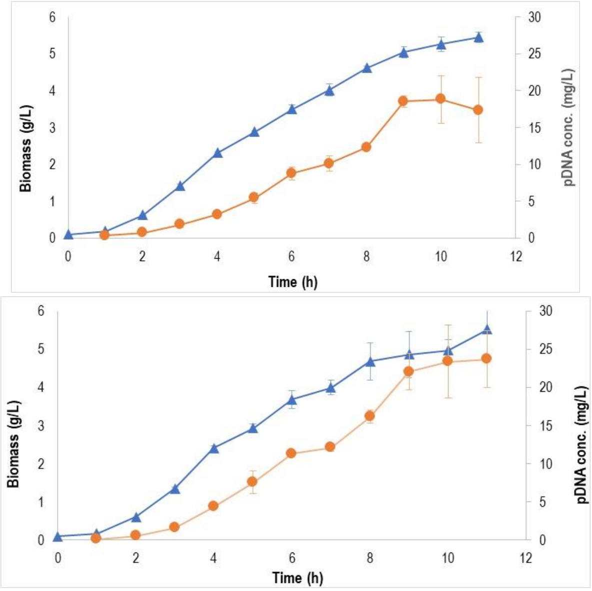

Figure S3. Time profiles of cultures of the wild type (left) and PFC (right) strains in Terrific Broth. Triangles: biomass; circles: plasmid DNA. Error bars show the standard deviation between triplicate experiments.

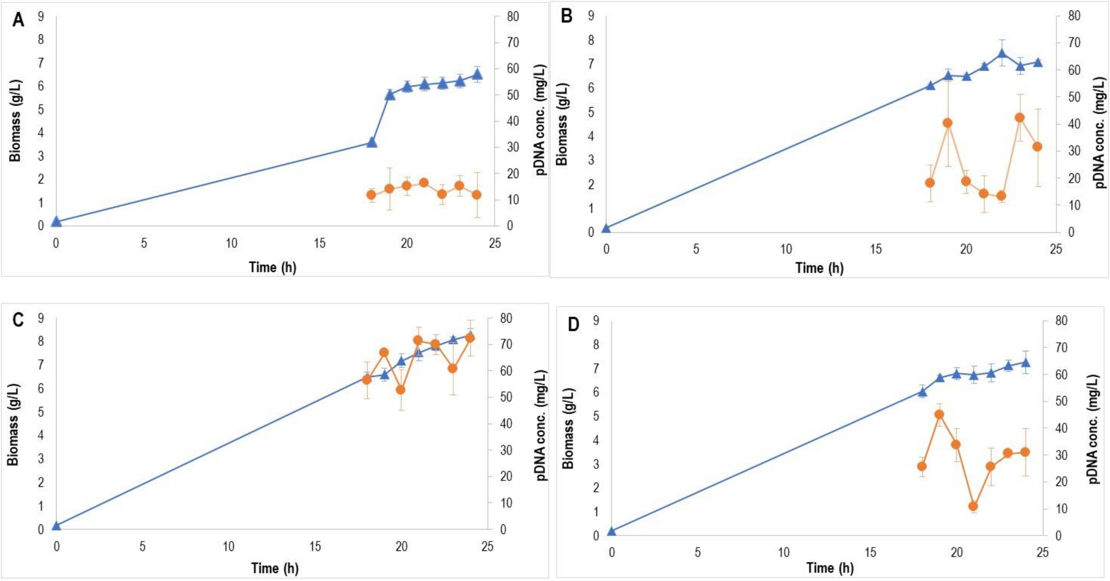

Figure S4. Time profiles of cultures of the wild type (A),wild type Δ*recA* (B), PFC (C) and PFC Δ*recA* (D) strains in Enpresso B Plasmid medium. Triangles: biomass; circles: plasmid DNA. Error bars show the standard deviation between triplicate experiments.

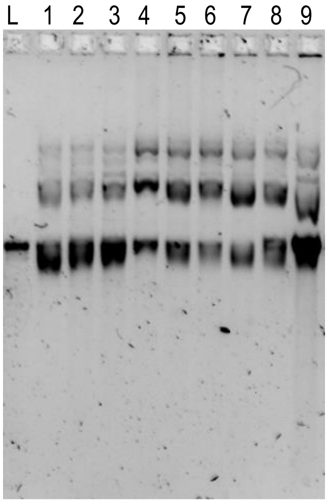

Figure S5. Example of agarose gel used for densitometric analysis of the pDNA produced by the proteome-reduced strain. L: pUC57Kan digested with *Bam*HI; 1-3: pDNA samples from triplicate cultures in LB; 4-6: pDNA samples from triplicate cultures in mineral medium; 7-9: pDNA samples from triplicate cultures in EnPresso B Plasmid medium.
